# Multi-Color Space Network for Salient Object Detection

**DOI:** 10.3390/s22093588

**Published:** 2022-05-09

**Authors:** Kyungjun Lee, Jechang Jeong

**Affiliations:** Department of Electronics and Computer Engineering, Hanyang University, Seoul 04763, Korea; kjlee888@hanyang.ac.kr

**Keywords:** salient object detection, multi-color space learning, fully convolutional network, atrous spatial pyramid pooling module, attention module

## Abstract

The salient object detection (SOD) technology predicts which object will attract the attention of an observer surveying a particular scene. Most state-of-the-art SOD methods are top-down mechanisms that apply fully convolutional networks (FCNs) of various structures to RGB images, extract features from them, and train a network. However, owing to the variety of factors that affect visual saliency, securing sufficient features from a single color space is difficult. Therefore, in this paper, we propose a multi-color space network (MCSNet) to detect salient objects using various saliency cues. First, the images were converted to HSV and grayscale color spaces to obtain saliency cues other than those provided by RGB color information. Each saliency cue was fed into two parallel VGG backbone networks to extract features. Contextual information was obtained from the extracted features using atrous spatial pyramid pooling (ASPP). The features obtained from both paths were passed through the attention module, and channel and spatial features were highlighted. Finally, the final saliency map was generated using a step-by-step residual refinement module (RRM). Furthermore, the network was trained with a bidirectional loss to supervise saliency detection results. Experiments on five public benchmark datasets showed that our proposed network achieved superior performance in terms of both subjective results and objective metrics.

## 1. Introduction

The amount of visual information received through the human visual system (HVS) for a certain period exceeds the amount of information that the human brain can process [1]. Therefore, the HVS assigns importance to perceived objects according to the visual information provided and focuses on the highly important ones. Objects on which human attention is focused in this process are called salient objects. The salient object detection (SOD) method uses a computational model that detects salient objects in an image by emulating the selective visual attention mechanism in humans. Its application can improve the performance of various types of computer vision such as image/video segmentation [2], image retrieval [3], object tracking [4], image classification [5], and video compression [6,7]. Accordingly, it is primarily applied in the pre-processing stage.

Visual attention operates according to different mechanisms: bottom-up and top-down. During the advent of saliency detection research, studies primarily focused on bottom-up attention mechanisms. Stimuli received by humans from a given scene through the eyes compete with each other while being transmitted from the bottom to the top regions of the brain [8]. Thus, the bottom-up process can also be referred to as a “data-driven” or “stimulus-driven” process. Wolfe [9] presented a variety of low-level cues or pre-attention features reflecting competition that can be acquired prior to the feature integration step. Most bottom-up saliency detection methods use these features to detect the saliency of a scene [10,11,12,13,14].

Meanwhile, the top-down attention mechanism is a volitional or mandatory response that occurs voluntarily to visual information with a specific goal [1,8,15,16]. Voluntary response is called a “task-driven” or “goal-driven” process because it discriminates visual information subjectively and is driven by a specific goal. Mandatory response is called a “knowledge-based” process because it biases visual information using prior knowledge derived from past experience, and it works even in the absence of subjective will. Recently, “knowledge-based” methods have received more attention than “task-driven” methods. This is because the development of deep learning technology has enabled the implementation of knowledge-based processes Moreover, detection methods using deep convolutional neural networks (CNNs) [17] exhibit excellent saliency detection.

Among CNN-based methods, fully convolutional networks (FCNs) [18] have been widely studied in recent years, and several SOD methods have been proposed based on them [19,20,21,22,23,24,25]. An FCN is an end-to-end network with a pyramid-like structure. From the input image, the FCN extracts low-level features from the shallow layers and high-level features from the deeper layers. Subsequently, the features extracted from each layer are fused to obtain contextual information, defining what the entire network is trained to detect:salient objects in images.

In conventional bottom-up techniques, researchers manually select the features to be used for saliency detection. The feature most representative of saliency feature is color. In any case, other features such as orientation, position, and shape are also used. In contrast, the FCN-based top-down technique extracts features required for SOD across multiple layers applied to the input image. Therefore, researchers do not need to manually select features. However, the SOD network is trained using only a dataset comprising images from the RGB color space unless pre-processing is performed.

In general, the simplest way to improve the detection performance of a network is to increase the number of layers in the backbone or the number of filters in each layer. This serves to obtain more features required for detection by further subdividing the characteristics of the salient object. However, these attempts have only been made on images in the RGB color space received as input from the network. Although the features obtained from the RGB channel povide important information to determine saliency, more diverse cues are involved in the process of recognizing a salient object according to the feature integration theory (FIT) [26]. Therefore, the network must be trained on the characteristics of salient objects by extracting features from various input cues and combining them.

To improve SOD performance by considering multiple cues contributing to visual attention, we propose a multi-color space network (MCSNet). First, the input RGB images are converted to the HSV and grayscale color space to obtain additional cues relating to saturation and luminance. The RGB channels with color information and the channels with information on saturation and luminance of the scene are input to the backbone network based on VGG-16 [27], and the features are extracted in parallel. An atrous spatial pyramid pooling (ASPP) [28] module is applied to the features output from each level of the VGG network to obtain spatial information. The enriched spatial information of saliency cue channels extracted by the ASPP is then weighted by the attention module [29,30,31] according to the predetermined characteristics. Finally, a saliency map is generated by further recovering the local details through a residual refinement module (RRM).

The primary contributions of this study are summarized below:

- An MCSNet was developed to achieve more accurate top-down saliency detection. In contrast to conventional methods that only use RGB color cues to learn the characteristics of salient objects, HSV and grayscale color spaces were utilized to leverage the information provided by various saliency cues. The VGG-based backbone network was divided into two parallel paths to extract features from RGB channels as well as channels with saturation and luminance information.
- Contextual information was obtained from the features extracted from the two backbone networks using the ASPP module. In addition, the attention module was applied to classify information according to the importance of features or spatial locations extracted from the color, saturation, and luminance information of the image. Features extracted from each level of the backbone network were mutually fused to create a final saliency map using RRM. Furthermore, bidirectional loss function was implemented to supervise the generation of the final saliency results.
- Five public salient object detection benchmarks were used in the experiment. Experimental results demonstrated that our proposed method achieved superior or comparable performance to the state-of-the-art methods.

## 2. Related Works

During the advent of saliency detection, low-level or handcrafted features that can be obtained from images were used to model human cognitive processes. These features include color [10,32,33], intensity [10], orientation [10], location [34], motion [35], horizontality [36], wavelet [37], curvature [38], spatial resolution [39], optical flow [40], symmetry [41], and texture contrast [42]. Low-level features are the most intuitive stimuli acquired by the HVS. Consequently, most saliency detection methods developed to implement the bottom-up recognition process use low-level features.

Recently, owing to the development of deep learning, saliency detection methods have significantly improved. Deep learning techniques are advantageous because they learn features from multiple images and use them to infer salient regions or objects from new images. Therefore, it has been actively used in research on top-down saliency detection methods that have largely been limited in the past. In particular, Long et al. [18] first presented the potential of pixel-to-pixel prediction networks for semantic segmentation by proposing an end-to-end FCN. Various FCN-based methods have been proposed since the advent of FCN. Deng et al. [43] proposed R${}^{3}$Net that progressively improves the saliency map by alternating low-level features and high-level features using a residual refinement block. Hu et al. [44] proposed RADF that aggregates the features of each layer in an iterative manner, with multiple levels of deep features, to produce distinct features that contain both the semantics and the details of the salient. Chen et al. [45] proposed RANet that applies residual learning into a holistically-nested edge detection (HED) [46] architecture and inverse attention to guide residual learning to discover missing object parts and residual details. Wu et al. [47] proposed CPD to improve performance by discarding low-level features and utilizing the generated relatively accurate attention maps to enhance high-level features. Zhao et al. [30] proposed PFANet, which applies a spatial attention module to low-level features and a context-aware pyramid function extraction module and channel-specific attention module to high-level features.

Unlike the method of integrating the context domain as a whole for saliency detection, methods for integrating the multi-scale context of the U-Net [48] architecture using various network modules have also been proposed. Zhang et al. [20] proposed Amulet that integrates features extracted from multilevel networks into the resolution of each level, combines features at each level, and subsequently predicts the saliency map in a recursive manner. Wang et al. [49] proposed a DGRL that localizes the salient object by iteratively focusing on the spatial distribution and refines the saliency map by the relationship between each pixel and its neighbors. Zhang et al. [50] proposed PAGR, which selectively integrates multiple contextual information of multi-level features using multi-path recurrent feedback that transfers global semantic information from the top layer to the shallower layers. Liu et al. [21] proposed PiCANet that improves the coarse saliency map by connecting features rich in spatial detail from the lower layers with features in the upper layer. Qin et al. [51] proposed a BASNet that consists of a deeply supervised encoder–decoder and residual refinement module. Liu et al. [22] proposed the PoolNet that improves saliency detection results through edge detection while extending the role of pooling based on the U-Net architecture. Chen et al. [52] proposed GCPANet that improves the relationships among different salient regions integrating low-level details, high-level semantic information, and global contextual information in an interweaved way.

The abovementioned deep learning-based top-down methods take RGB images as input and extract features necessary for saliency detection using a convolutional layer. Meanwhile, detection methods for images from color spaces other than RGB have been proposed. These methods use RGB-D data to which the depth information of an image is added as an additional cue, which is required to generate the saliency map of images. Qu et al. [53] proposed a DF using deep learning technology for the first time for RGB-D-based SOD tasks. DF derives saliency confidence values through the CNN architecture from RGB-D data and subsequently integrates the superpixel-based Laplacian propagation framework with the trained CNN to generate final detection results. Han et al. [54] proposed a CTMF that utilizes a CNN to learn high-level features from RGB and depth images. In addition, it complements the depths modalities by integrating the structure of color networks into them. The DMRA proposed by Piao et al. [55] and the MMCI proposed by Chen et al. [56] have a two-stream architecture that applies the same backbone network to RGB images and depth data. Color and depth features extracted through backbone complement each other through additional feature integration methods to create the final saliency map.

Maximizing the number of acquired features is essential for improving the accuracy of saliency detection. RGB-D-based methods accordingly use depth information in addition to image color. Moreover, various methods for designing deep learning networks to exploit these extra sources of information have been developed. Furthermore, considering that salient objects are prioritized by observers, they are probably located in the front region of the image. Thus, salient objects correspond to the foreground, and the rest of the image can be considered as the background. Therefore, the depth map is useful for detecting salient objects. However, a special device such as a Kinect is required to obtain a depth map. In addition, the failure area formed during the generation of the depth map must be filled or disparity correction must be performed.

To implement a deep learning-based SOD network that receives multiple features as input, we proposed an MCSNet using easy-extractable features that are conducive to saliency prediction. It converts the original RGB image into HSV and grayscale color spaces that provide information other than color. Backbone networks extract low-level to high-level features from different color space inputs in parallel. The features extracted from the two streams are combined while considering global characteristics. The two streams learn global characteristics in a manner that complements each other’s information according to the level of the backbone. Finally, the network is subjected to a refinement process to generate the final saliency map.

## 3. Proposed Methodology

**Figure 1:**
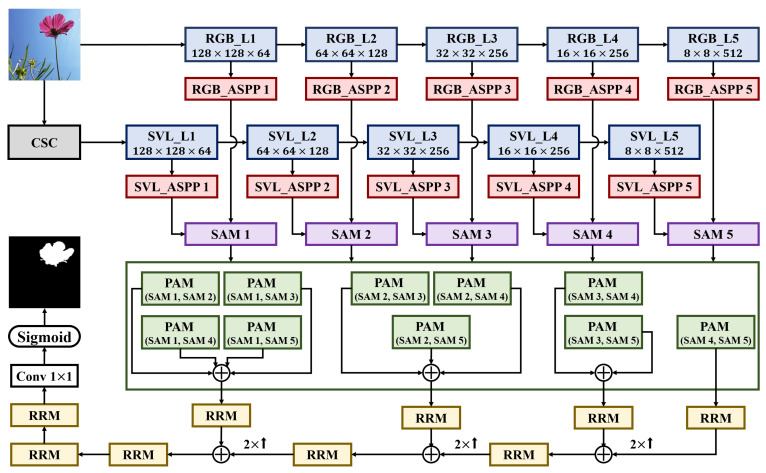
Overall architecture of the proposed MCSNet. CSC represents a color space converter for creating additional saliency cues from the input image. L1-L5 represent each level of the backbone network modified based on the VGG network. ASPP represents atrous spatial pyramid pooling. SAM and PAM represent serial attention module and parallel attention module, respectively. RRM represents residual refinement module. The various ⊕ denote the element-wise summation module.

In this section, we introduce details of the proposed MCSNet as shown in Figure 1. We focus on the components of MCSNet: color space converter (CSC), backbone network based on VGG, ASPP, the two types of attention modules, and RRM. Finally, we introduce the loss function developed to strictly supervise the saliency map results generated by the MCSNet.

### 3.1. Preprocessing for Additional Saliency Cues

**Figure 2:**
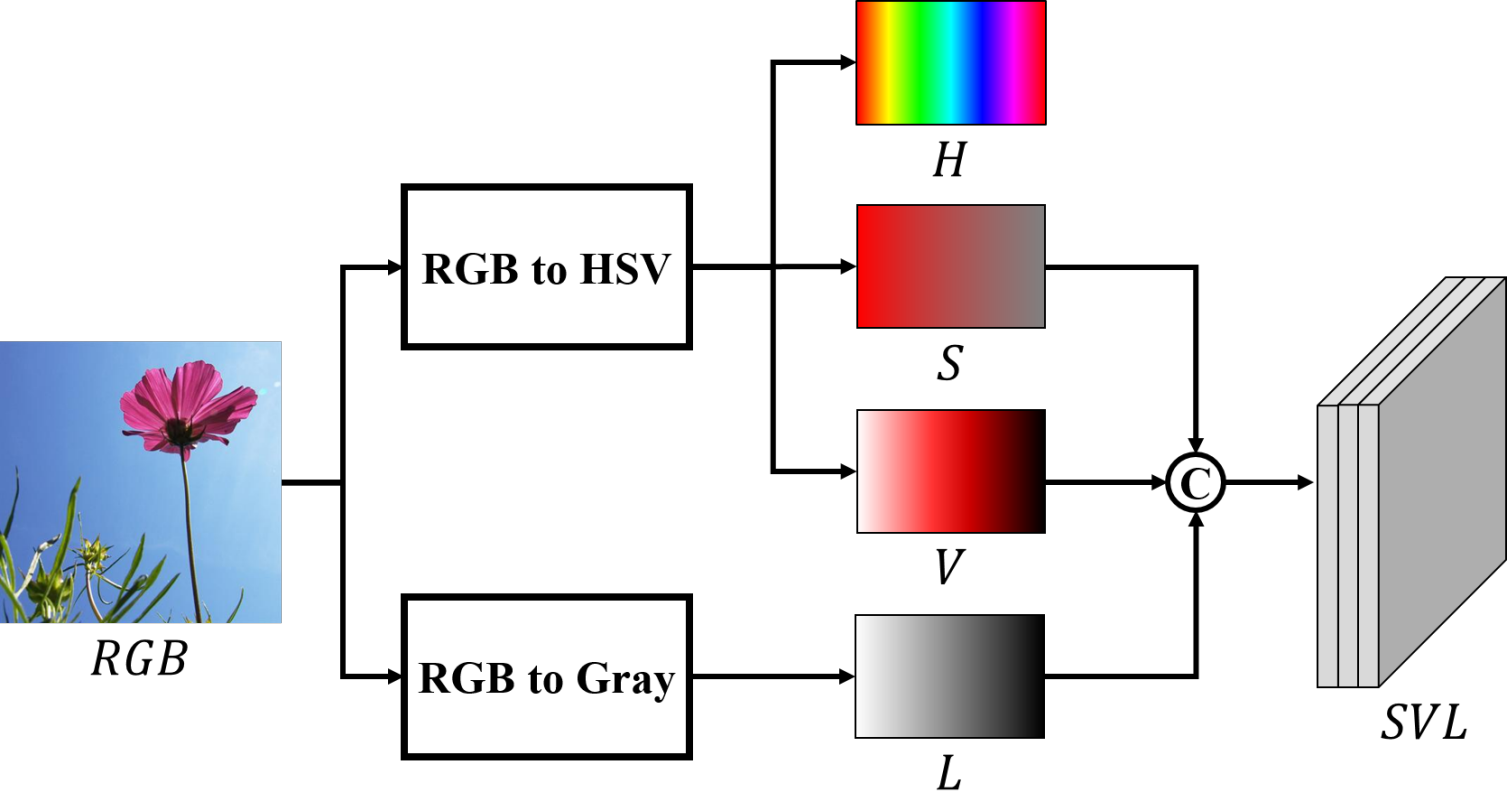
Structure and details of the CSC module. “C” denotes the concatenation module.

To create additional saliency cues for use in SOD other than those from the RGB color space, we first applied CSC as a preprocessor to transform the color space. The overall process followed by the CSC is shown in Figure 2.

First, the input RGB image is converted into the HSV color space to obtain the saturation and value components for use as additional saliency cues. The *H*, *S*, and *V* represent the hue, saturation, and value channels, respectively, and are calculated as follows, where *R*, *G*, and *B* represent the red, green, and blue channels, respectively, normalized to the range of [0, 1].

(1) $$\begin{array}{c} C_{max}=\max\left(R,G,B\right),\;\;\;\;\;\;C_{min}=\min\left(R,G,B\right),\;\;\;\;\;\;\Delta =C_{max}-C_{min},\\ \begin{array}{cc} H & =\left\{\begin{array}{cc} 60^{\circ }\times \left(\frac{G-B}{\Delta }\right)\;\mathrm{mod}\;6 & \;\mathrm{if}\;C_{max}=R\\ 60^{\circ }\times \left(\frac{B-R}{\Delta }\right)+2 & \;\mathrm{if}\;C_{max}=G\\ 60^{\circ }\times \left(\frac{B-R}{\Delta }\right)+4 & \;\mathrm{if}\;C_{max}=B \end{array}\right.\\ S & =\left\{\begin{array}{cc} \;\;\;\;0 & \;\mathrm{if}\;C_{max}=0\\ \frac{\Delta }{C_{max}} & \;\mathrm{if}\;C_{max}\neq 0 \end{array}\right.\\ V & =C_{max} \end{array} \end{array}$$

Next, the input RGB image is converted to a grayscale color space to obtain the luminance component. Luminance *L* is calculated as follows, according to the ITU-R BT.709 specification [57].

(2) L=0.2125R+0.7154G+0.0721B

Finally, *SVL* channels are created by concatenating the *S*, *V*, and *L* channels computed through Equations (1) and (2).

**Figure 3:**
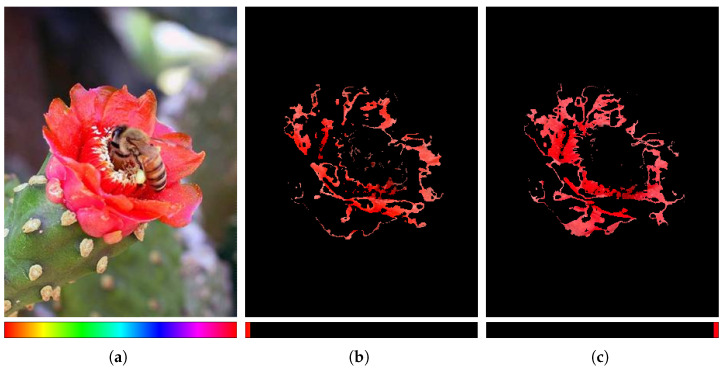
Example of why the hue channel of the HSV color space must be excluded. The images in the first row are RGB images corresponding to the hue spectrum normalized to the range [0, 1] shown in the second row: (**a**) original image in range [0, 1]; (**b**) RGB image corresponding to the range [0, 0.02]; (**c**) RGB image corresponding to the range [0.98, 1].

There are two reasons for excluding a hue channel from network training. The first is that the RGB color space and the hue channel possess overlapping color information. The second is the discontinuity of the hue component that can be seen in Figure 3. Figure 3a shows the original RGB image and the hue spectrum normalized to [0, 1]. Furthermore, hue is expressed as an angle relative to red on the color wheel based on the Munsell color system [58,59]. Thus, red-based colors are distributed around the minimum and maximum values of the spectrum. Therefore, although the flower in Figure 3a is colored red throughout, different regions of the flower are divided by the values at both ends of the hue spectrum as shown in Figure 3b,c. This discontinuity in a particular color can interfere with the training of the filter to extract the features. Thus, the hue channel is excluded from the process of acquiring additional saliency cues.

### 3.2. Backbone

Despite its simple structure, VGGNet can extract all low- and high-level features required for the image recognition process. Because of these advantages, it is actively used in SOD. We adopted the modified VGG-16 structure for MCSNet as the backbone network for extracting features from saliency cues. Our modified structure comprises only five levels that remove the fully connected (FC) layer located behind the conv layer of the existing VGG-16 network. Detailed parameter settings such as the size of the image input to the network and the channel of each conv layer are shown in Table 1. The backbone networks function parallell to the extract features from the original RGB color space as well as the SVL channel generated through CSC as shown in Figure 1. Both the backbone networks have the same structure and parameter settings. Finally, the features created as a result of the last conv layer at each level of the backbone network are fed into the ASPP module.

### 3.3. ASPP Module

FCN acquires global semantic information of images by reducing the size of features through pooling. However, local information is lost owing to the reduced feature resolution. Therefore, the ASPP module is applied to provide more contextual and local information that might be lost in each level.

**Figure 4:**
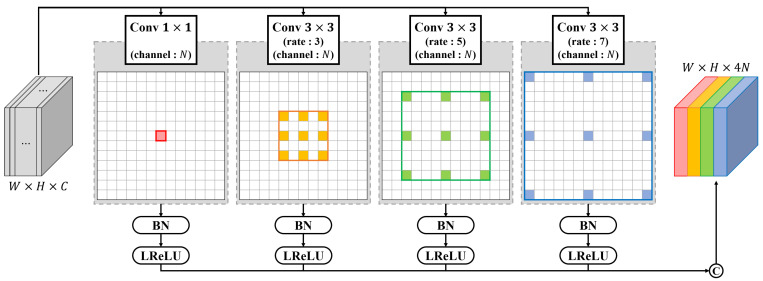
Structure and details of the ASPP module. BN and LReLU represent batch normalization and leaky rectified linear units, respectively. “C” denotes the concatenation module.

The ASPP module applied to MCSNet comprises one 1×1 conv and three 3×3 dilated convs with rates of 3, 5, and 7, respectively, as shown in Figure 4. The width, height, and channels of a feature are denoted by *W*, *H*, and *C*, respectively, and the four convs are performed in parallel to extract *N* features each. Subsequently, the features activated through BN and LReLU are concatenated. Thus, it is possible to even obtain secure features. This is enabled by the contextual correlation derived from the pixel-wise spatial information of the features that are extracted from each level of the backbone and a wider receptive field.

### 3.4. Two Types of Attention Modules

The output generated by the CNNs comprises multiple feature channels, and the factor that affects the saliency map generation differs with the input image. Moreover, the local information differs according to the depth of the network or feature channels. Therefore, we used two types of attention modules to highlight the characteristics of features that improve SOD performance.

**Figure 5:**
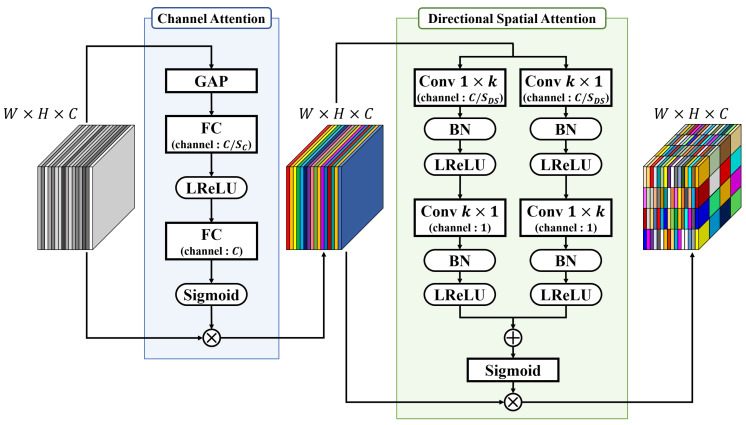
Structure and details of the serial attention module (SAM). ⨂ and ⨁ denote the element-wise multiplication and summation module, respectively.

The first type is the SAM shown in Figure 5 that is applied to the output of the ASPP module. In SAM, the channel attention [60] and directional spatial attention modules [30,61] that extract the global correlation, while considering the directionality, are sequentially performed.

The result generated by the ASPP module is a concatenation of the information extracted for each of the four kernel sizes. The channel attention module determines which channel is to be prioritized among the channels containing different regional information according to the depth of the backbone network. First, the input features generate a vector of channel size *C* that represents each channel of the feature through global average pooling (GAP). Subsequently, the vector is converted into a latent vector, in which useful information is compressed through an FC layer. This reduces the channel size to $C/S_{C}$ using the squeeze parameter $S_{C}$. A latent vector activated with LReLU becomes a scaling vector using an FC layer with a channel size of *C* and a Sigmoid. Finally, the input feature is multiplied by the scaling vector to output the feature with the channel emphasized according to the degree of contribution to the SOD.

The directional spatial attention module activated after the channel attention module considers the directionality. Therefore, it considers the boundary between the salient object and the background and assigns weights according to wider regional correlations. First, as a receptive field considering directionality, the horizontal and vertical 1×k conv and k×1 conv are parallelly applied to the input features. The size of the output channel is reduced to $C/S_{DS}$ using the squeeze parameter $S_{DS}$. Subsequently, k×1 conv and 1×k conv of vertical and horizontal shapes are applied to the resulting features to output features with one channel each. BN and LReLU are applied after every conv layer. The two channels are summed element-wise and activated using a Sigmoid to capture directional spatial concerns. Finally, spatial information is emphasized by element-wise multiplication of input features.

The second type is a PAM. The features from the shallower levels of the backbone network contain local and detailed information of the image owing to the small receptive field compared with the image size. In contrast, the feature in the deeper levels are smaller in size due to pooling; thus, the receptive field can handle a wider area of correlation in the deeper levels than in the shallow levels. Therefore, the features contain semantic and global information of the image. To complement this imbalanced information provided by the features, PAM is performed for all cases that can be paired with the five SAM result features generated at each level of the backbone network as shown in Figure 1.

**Figure 6:**
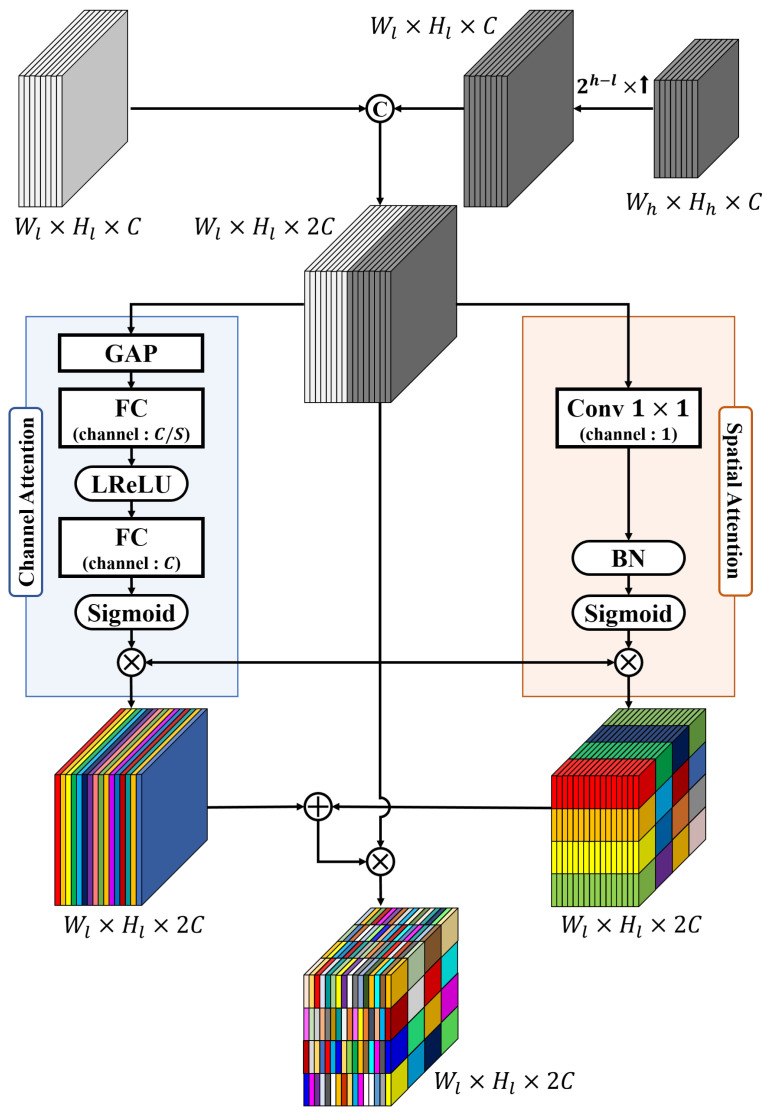
Structure and details of the parallel attention module (PAM).

The structure of PAM is shown in Figure 6. When $l\in \left\{1,2,3,4\right\}$, $h\in \left\{2,3,4,5\right\}$, l<k, the *l*-th low-level SAM feature and the *h*-th high-level SAM feature are input to the PAM. To concatenate the two features, the width, $W_{h}$, and height, $H_{h}$, of the high-level feature are upsampled by $2^{h-l}$ times to match the width, $W_{l}$, and height, $H_{l}$, of the low-level feature. Both features have an identical number of channels as *C*; thus, the concatenated feature has the shape $W_{l}\times H_{l}\times 2C$. Subsequently, the channel attention module and spatial attention module are performed in parallel on the concatenated feature. Channel attention modules have the same structure as that of the SAM. To highlight and scale pixel-wise local information, the spatial attention module multiplies the spatial information collected by 1×1 conv to input features, similar to the structure proposed by SCA-CNN [62]. The results of the two attention modules are summed element-wise, and the concatenated features are multiplied element-wise to finally obtain a channel-wise and spatially emphasized feature.

### 3.5. RRM

**Figure 7:**
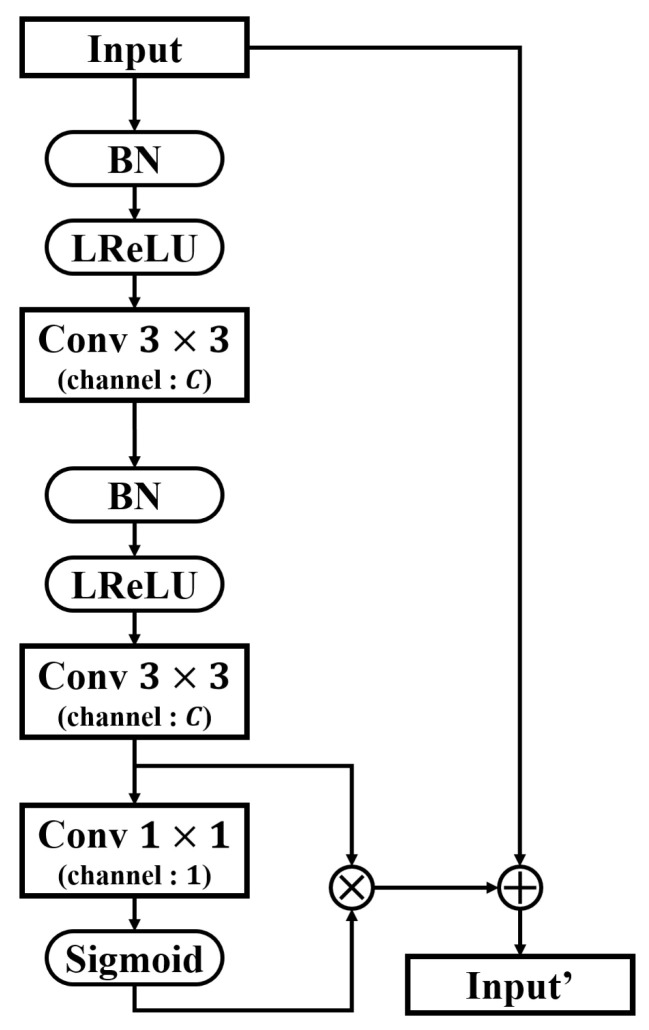
Structure and details of the RRM.

The various features extracted and scaled from low- to high-level from the saliency cues of the image must be integrated to predict the salient object. To achieve effective integration, we used the feature fusion network structure and RRM proposed by CAGNet [63]. RRM is a residual block in which spatial attention modules are added to two 3×3 conv layers, as shown in Figure 7. Subsequently, the input features are skip-connected to the result. The primary difference from the general residual block [64] is that BN and LReLU are performed before two 3×3 conv layers, which are initially performed according to the full preactivation structure as demonstrated in [63,65]. The RRM learns the residuals between input features, and the output passes through the conv layer to emphasize the salient region of the output feature and suppress the coarse region.

### 3.6. Bidirectional Loss Function

The ground truth images of almost all datasets used for SOD were binary images in which salient objects and backgrounds were denoted using 1 and 0, respectively. Thus, the cross-entropy loss function was adopted to supervise the training of the network for SOD. Assuming that the ground truth corresponding to the input image and the predicted saliency map generated by the network are *G* and *S*, respectively, the cross-entropy loss $L_{ce}$ is defined as follows:

(3) $$L_{ce}\left(G,S\right)=-\sum_{\left(x,y\right)}^{}\left[\omega_{p}G\left(x,y\right)logS\left(x,y\right)+\left\{1-G\left(x,y\right)\right\}log\left\{1-S\left(x,y\right)\right\}\right],$$

where $\left(x,y\right)$ is the position of the pixel, and $\omega_{p}$ is a weighting parameter that adjusts the cost of positive errors relative to negative errors.

To detect salient objects in the image more accurately, a small loss must occur in both the salient and background parts. However, the cross-entropy loss is sensitive only to the saliency class and not to the background class. Therefore, we adopted a modified loss function, where both the salient and background parts have the same loss function sensitivity. The proposed bidirectional loss function *L* is formulated as follows:

(4) $$L=\alpha_{1}L_{ce}\left(G_{F},S_{F}\right)+\alpha_{2}L_{ce}\left(G_{B},S_{B}\right),$$

where $\alpha_{1}$ and $\alpha_{2}$ are the loss weights used to balance the foreground and background loss terms, respectively. To give equal weight to the foreground and background losses, we set $\alpha_{1}$ and $\alpha_{2}$ to 0.5. $G_{F}$ and $S_{F}$ are the ground truth of the foreground and saliency map output from the networks, respectively, and they are the same as *G* and *S*. $G_{B}$ and $S_{B}$ are the ground truth and saliency maps of the background that are obtained by inverting $G_{F}$ and $S_{F}$ as follows:

(5) $$\begin{array}{cc} G_{B} & =1-G_{F}\\ S_{B} & =1-S_{F}. \end{array}$$

## 4. Experiments

### 4.1. Datasets

The proposed method was evaluated on five public SOD datasets. The characteristics of each dataset are as follows:

DUT-OMRON [66] contained 5168 images with one or more salient objects and a relatively complex background. DUTS [67] contained 15,572 images of which 10,553 images were used for training, and 5019 images were used for testing. All the training images were collected from ImageNet DET training/validation sets [68], while test images were collected from the ImageNet DET test set [68] and the SUN [69] dataset. ECSSD [70] comprised 1000 images obtained from the Internet, typically containing natural images. The selected images included semantically meaningful but structurally complex backgrounds. HKU-IS [71] contained 4447 challenging images, most of which had either low contrast or multiple salient objects. PASCAL-S [72] was built on the validation set of the PASCAL VOC 2010 segmentation challenge. It contained 850 natural images with multiple objects in a scene [73,74].

### 4.2. Evaluation Metrics

For objective performance evaluation, we adopted three popular metrics, including Precision-recall (PR) curve, F-measure, and mean absolute error (MAE).

The PR curve plots precision on the *y*-axis and recall on the *x*-axis for different probability thresholds. Precision (also known as the positive predicted value) is the ratio of the correctly predicted salient regions to all predicted salient regions. Recall (also known as the true positive rate or sensitivity) is the ratio of the correctly predicted salient region to the actual salient region. Precision and recall were calculated using the following equations:

(6) $$precision=\frac{TP}{TP+FP},$$

(7) $$recall=\frac{TP}{TP+FN},$$

where TP, FP, and FN are the true-positive, false-positive, and false-negative rates, respectively.

The F-measure is the weighted harmonic mean of precision and recall. It was adopted to measure the overall performance of the saliency detection model and was calculated as follows:

(8) $$F_{\beta }=\frac{(1+\beta^{2})precision\times recall}{\beta^{2}\times precision+recall},$$

where the weighting parameter $\beta^{2}$ was set to 0.3 for our implementation.

MAE is a measure of errors between paired observations expressing the same phenomenon. In our study, we calculated the average difference between the predicted saliency map *S* and ground truth image *G*. Subsequently, MAE was calculated as follows:

(9) $$MAE=\frac{1}{W\times H}\sum_{y=1}^{H}\sum_{x=1}^{W}\left|S\left(x,y\right)-G\left(x,y\right)\right|,$$

where *W* and *H* are the width and height of the image, respectively, and (x,y) is a pixel coordinate.

### 4.3. Implementation Details

The proposed MCSNet was implemented on the Keras (https://keras.io/ accessed on 5 May 2022) framework using TensorFlow (https://www.tensorflow.org/ accessed on 5 May 2022) backend. We conducted our experiments on an Intel Core i7-7700 3.60 GHU using an NVIDIA GeForce RTX 3080Ti GPU (12 G). We randomly selected 80% of all images in the dataset introduced in Section 4.1 for the training set and the remaining 20% as the validation set. All training and ground truth images were resized to 128×128 pixels. Our model was trained for 100 epochs using the Adam optimizer [75]. The initial learning rate was set to 0.001, which decreased by 50% when the validation accuracy plateaued. The batch size and negative slope coefficient for the LReLU were set to 8 and 0.05, respectively.

### 4.4. Comparison with State-of-the-Art Methods

MCSNet was compared with 13 state-of-the-art methods, including Amulet [20], DGRL [49], PAGR [50], PiCANet [21], R${}^{3}$Net [43], RADF [44], RANet [45], BASNet [51], CPD-ResNet50 [47], CPD-VGG16 [47], PoolNet [22], PFANet [30], and GCPANet [52]. To ensure fair comparison, the saliency map published by the author who proposed each saliency detection method was used. In the absence of published data, the results were output through the network trained using the parameters set by each author.

#### 4.4.1. Visual Comparison

**Figure 8:**
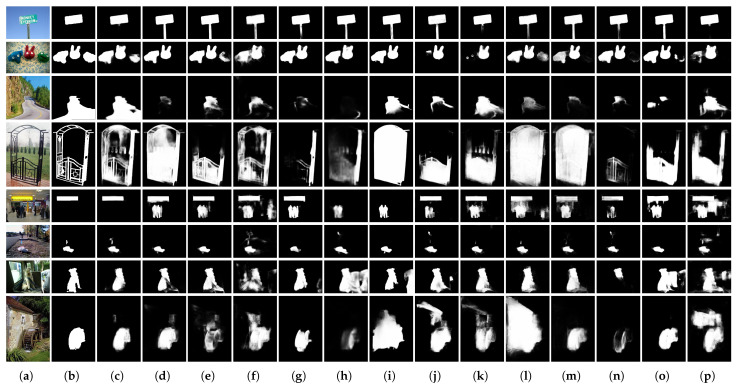
Visual comparison between our MCSNet and 13 state-of-the-art methods: (**a**) original input image; (**b**) ground truth; (**c**) our MCSNet; (**d**) GCPANet [52]; (**e**) PoolNet [22]; (**f**) PFANet [30]; (**g**) CPD-VGG16 [47]; (**h**) CPD-ResNet50 [47]; (**i**) BASNet [51]; (**j**) RANet [45]; (**k**) RADF [44]; (**l**) R${}^{3}$Net [43]; (**m**) PiCANet [21]; (**n**) PAGR [50]; (**o**) DGRL [49]; and (**p**) Amulet [20].

A visual comparison of the results is presented in Figure 8. It considers images illustrating scenarios of varying complexity: from simple situations to challenging highly difficult ones. The first and second rows show the results in a situation in which the foreground and background of the image are distinct. In the first row, most state-of-the-art methods detected all signs and poles that were clearly distinct from the background, whereas MCSNet excluded the poles. In the second row, it detected not only the red and blue dolls, but also the green doll located on the right. Conversely, the third peak at a row corresponds to a case where the distinction between foreground and background was ambiguous. Here, MCSNet effectively distinguished objects that occupied a significant portion from the center to the bottom of the video. The fourth row shows results for images including objects with complex details. In the images shown in the fifth row, the background was suppressed, and salient objects were emphasized in consideration of the global context in a situation where multiple objects exist. The last three rows show the results of MCSNet surpassing existing methods in images of complex scenarios in which various contents with similar or competitive characteristics to salient objects exist in the background. Thus, we can conclude that MCSNet performs well in difficult situations.

#### 4.4.2. Quantitative Comparison

Quantitative comparison results in terms of MAE and F-measure between MCSNet and 13 state-of-the-art methods are listed in Table 2. MCSNet performed best on the DUT-OMRON dataset with one or more salient objects and a relatively cluttered background. The ECSSD and PASCAL-S datasets exhibited the highest performance after GCPANet. In the case of the DUTS and HKU-IS datasets, the MAE of MCSNet had the fourth best results, whereas its F-measure was among the top three results.

**Figure 9:**
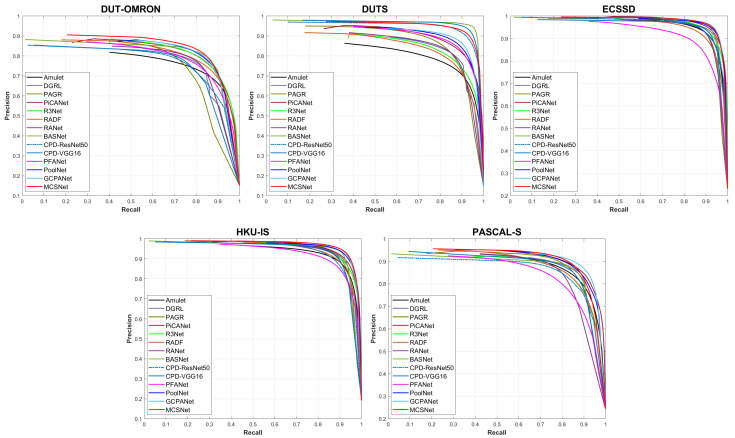
PR curves on five representative datasets.

Figure 9 shows the PR curves for the five datasets. It can be seen that the curve of MCSNet is placed on top of most of the other curves. From this PR curve, we can conclude that the proposed method is generally superior to the other state-of-the-art methods, especially in DUT-OMRON. Thus, the proposed method can effectively compete with existing methods, indicating that the method of detecting salient objects by extracting image features from various saliency cues is effective.

## 5. Conclusions

In this paper, we proposed a network called MCSNet to detect salient objects in images using various saliency cues. We noted that an observer classifies an object as salient based on factors other than color. Thus, in addition to the RGB color space of the original input image, an SVL color space was developed by concatenating the saturation and value components of HSV and the luminance components of grayscale. Two input channels were input to two parallel backbone networks based on VGG-16, and the relevant features were extracted. The ASPP module was applied to improve the local information of the features extracted from the backbone, and two types of attention modules were introduced to improve feature expression. Moreover, a final saliency map was generated by aggregating features while improving semantic information using RRM. Furthermore, the SOD performance of several methods on five published datasets were experimentally evaluated. The results showed that the proposed method was superior to most of the existing state-of-the-art methods in terms of various evaluation metrics. This indicates that detecting salient objects using features extracted from various saliency cues is effective for the network design of top-down SOD.

However, this study had a few limitations. The only components other than RGB used in this study were HSV and luminance; more accurate detection can be achieved if more saliency cues are included. Moreover, the two parallel backbone networks used possessed identical structures. However, backbone networks with different structures should be used to better cater to the characteristics of each cue. Therefore, in our future work, we will focus on the study of extended SOD network design using multiple cues.

## Figures and Tables

**Table 1:** Parameter settings of the modified VGG-based backbone.

Level	Layer	Size		Channel		Kernel Size	Stride
		Input	Output	Input	Output		
L1	Conv1-1	128×128	128×128	3	64	3×3	1
	Conv1-2	128×128	128×128	64	64	3×3	1
	MaxPool	128×128	64×64			2×2	2
L2	Conv2-1	64×64	64×64	64	128	3×3	1
	Conv2-2	64×64	64×64	128	128	3×3	1
	MaxPool	64×64	32×32			2×2	2
L3	Conv3-1	32×32	32×32	128	256	3×3	1
	Conv3-2	32×32	32×32	256	256	3×3	1
	Conv3-3	32×32	32×32	256	256	3×3	1
	MaxPool	32×32	16×16			2×2	2
L4	Conv4-1	16×16	16×16	256	256	3×3	1
	Conv4-2	16×16	16×16	256	256	3×3	1
	Conv4-3	16×16	16×16	256	256	3×3	1
	MaxPool	16×16	8×8			2×2	2
L5	Conv5-1	8×8	8×8	256	512	3×3	1
	Conv5-2	8×8	8×8	512	512	3×3	1
	Conv5-3	8×8	8×8	512	512	3×3	1

Note: All convolutional layers used in the modified VGG are activated by leaky rectified linear unit (LReLU) function after batch normalization (BN).

**Table 2:** Quantitative comparison using five representative datasets in terms of the MAE and F-measure. The maxF designation denotes max F-measure for the best performance that a method can achieve. ↓ denotes that smaller is better, and ↑ denotes that larger is better. The best three results are labeled **Red**, **Blue**, and **Green**, in that order.

Methods	DUT-OMRON		DUTS		ECSSD		HKU-IS		PASCAL-S	
	MAE↓	maxF↑	MAE↓	maxF↑	MAE↓	maxF↑	MAE↓	maxF↑	MAE↓	maxF↑
Amulet [20]	0.0957	0.7537	0.0816	0.7835	0.0517	0.9254	0.0501	0.8991	0.0923	0.8527
DGRL [49]	0.0651	0.7827	0.0492	0.8324	0.0348	0.9356	0.0343	0.9198	0.0779	0.8649
PAGR [50]	0.0734	0.7790	0.0556	0.8530	0.0569	0.9331	0.0449	0.9230	0.0888	0.8712
PiCANet [21]	0.0655	0.8074	0.0495	0.8635	0.0405	0.9424	0.0419	0.9227	0.0783	**0.8788**
R${}^{3}$Net [43]	0.0707	0.8079	0.0646	0.8233	0.0466	0.9346	0.0449	0.9143	0.0947	0.8475
RADF [44]	0.0701	0.7918	0.0704	0.8138	0.0603	0.9161	0.0508	0.9060	0.1009	0.8470
RANet [45]	0.0613	0.7904	0.0579	0.8374	0.0499	0.9285	0.0452	0.9154	0.0968	0.8504
BASNet [51]	0.0556	0.8182	**0.0197**	**0.9499**	**0.0331**	0.9467	**0.0306**	0.9323	0.0795	0.8682
CPD-ResNet50 [47]	0.0636	0.7685	**0.0323**	0.9195	0.0409	0.9299	0.0437	0.9046	0.0851	0.8403
CPD-VGG16 [47]	0.0575	0.7757	**0.0226**	**0.9387**	0.0355	0.9332	0.0363	0.9186	0.0778	0.8609
PFANet [30]	0.0763	0.7801	0.0716	0.8677	0.0766	0.8816	0.0604	0.8853	0.1189	0.8173
PoolNet [22]	**0.0549**	**0.8183**	0.0400	0.8783	0.0332	**0.9468**	**0.0298**	**0.9338**	**0.0762**	0.8772
GCPANet [52]	**0.0553**	**0.8196**	0.0370	0.8865	**0.0308**	**0.9521**	**0.0295**	**0.9404**	**0.0638**	**0.8899**
**MCSNet**	**0.0518**	**0.8294**	0.0363	**0.9224**	**0.0322**	**0.9507**	0.0313	**0.9394**	**0.0723**	**0.8842**

## Data Availability

Not applicable.

## Abbreviations

The following abbreviations are used in this manuscript:

SOD	Salient object detection
FCN	Fully convolutional network
MCSNet	Multi-color space network
ASPP	Atrous spatial pyramid pooling
RRM	Residual refinement module
HVS	Human visual system
CNN	Convolutional neural network
FIT	Feature integration theory
HED	Holistically-nested edge detection
CSC	Color space converter
FC layer	Fully connected layer
LReLU	Leaky rectified linear unit
BN	Batch normalization
SAM	Serial attention module
GAP	Global average pooling
PAM	Parallel attention module
PR curve	Precision-recall curve
MAE	Mean absolute error
